# Light Drives and Temperature Modulates: Variation of Phenolic Compounds Profile in Relation to Photosynthesis in Spring Barley

**DOI:** 10.3390/ijms24032427

**Published:** 2023-01-26

**Authors:** Daniel Vrábl, Jakub Nezval, Radomír Pech, Adriana Volná, Petra Mašková, Jan Pleva, Nikola Kuzniciusová, Michaela Provazová, Michal Štroch, Vladimír Špunda

**Affiliations:** 1 Department of Physics, Faculty of Science, University of Ostrava, 710 00 Ostrava, Czech Republic; 2 Department of Experimental Plant Biology, Faculty of Science, Charles University, 128 00 Prague, Czech Republic; 3 Global Change Research Institute, Czech Academy of Sciences, 603 00 Brno, Czech Republic

**Keywords:** antioxidants, carotenoids, CO_2_ assimilation, flavonoids, gene expression, HPLC, non-structural carbohydrates, photosynthetically active radiation (PAR), secondary metabolism, spring barley (*Hordeum vulgare*), temperature

## Abstract

Accumulation and metabolic profile of phenolic compounds (PheCs; serving as UV-screening pigments and antioxidants) as well as carbon fixation rate (A_n_) and plant growth are sensitive to irradiance and temperature. Since these factors are naturally co-acting in the environment, it is worthy to study the combined effects of these environmental factors to assess their possible physiological consequences. We investigated how low and high irradiance in combination with different temperatures modify the metabolic profile of PheCs and expression of genes involved in the antioxidative enzyme and PheCs biosynthesis, in relation to photosynthetic activity and availability of non-structural carbohydrates (NSC) in spring barley seedlings. High irradiance positively affected A_n_, NSC, PheCs content, and antioxidant activity (AOX). High temperature led to decreased A_n_, NSC, and increased dark respiration, whilst low temperature was accompanied by reduction of UV-A shielding but increase of PheCs content and AOX. Besides that, irradiance and temperature caused changes in the metabolic profile of PheCs, particularly alteration in homoorientin/isovitexin derivatives ratio, possibly related to demands on AOX-based protection. Moreover, we also observed changes in the ratio of sinapoyl-/feruloyl- acylated flavonoids, the function of which is not yet known. The data also strongly suggested that the NSC content may support the PheCs production.

## 1. Introduction

Phenolic compounds (PheCs) represent a large class of secondary metabolites, with variable chemical structure and function. The accumulation of phenolic compounds in plants often occurs under a wide range of abiotic and biotic stress conditions. They act particularly as UV-screening pigments and as effective antioxidants, contributing to protection of leaves from damage by reactive oxygen species (ROS) [1,2]. Biosynthesis of PheCs involves a cascade of several biosynthetic pathways. Products of glycolysis and pentose phosphate cycle enter the shikimate pathway, while phenylalanine produced by the shikimate pathway is the precursor of phenylpropanoid metabolism which is used in specific flavonoid branch pathways [3] (Figure S1). Both glycolysis and pentose phosphate cycle are maintained by the availability of triose and hexose phosphates produced by photosynthesis and subsequent primary metabolism. Indeed, Lloyd and Zakhleniuk [4] observed that excessive accumulation of sucrose and other carbohydrates in *Arabidopsis thaliana* mutant *pho3* (harboring a defective copy of the *SUC2* gene, encoding a sucrose-proton symporter that is important for phloem loading of sucrose [5]), was accompanied by dramatic increases in the expression of genes for transcription factors and enzymes involved in biosynthesis of PheCs (including anthocyanins). The upregulation of PheCs biosynthesis, representing alternative sink for carbon, was found to be specifically responsive to sucrose, which acts as a signaling molecule shifting the carbon allocation from primary to secondary metabolites [6].

Irradiance and temperature are among the most important environmental drivers that influence the rate of photosynthesis, primary assimilates and biomass production. Irradiance is the main source of energy, which is later conserved within production of ATP and NADPH intended especially for CO_2_ assimilation in Calvin–Benson cycle and synthesis of primary assimilates including triose and hexose phosphates. Besides that, temperature affects primary metabolism due to changes in the photosystem II (PSII) function, electron transport rate, the rate of photorespiration, Rubisco activation, and variation of intercellular CO_2_ concentration due to stomata closure [7]. However, beyond their optimal range, both irradiance and temperature results in a decrease in photosynthetic activity and an increase in oxidative pressure on plant metabolism.

In addition to the direct effects of high photosynthetically active radiation (PAR) resulting from excessive excitation of photosynthetic apparatus, plants undergo physiological and biochemical adjustments of cellular homeostasis to face the disorders caused mainly by higher production of ROS. Exposure of plants to excess PAR radiation with harmful potential often leads to accumulation of PheCs, particularly flavonoids (FLVs). Phenolic compounds have several protective functions. PheCs located in the cuticular wax layer and within the epidermal cells contribute to protection of photosynthetic apparatus through shielding of UV A/B radiation and short wavelengths of PAR. In addition, leaves exposed to UV-B and high PAR preferentially synthesized ortho-dihydroxylated B-ring FLVs as compared with monohydroxy-substituted counterparts [1,8]. Particularly, ortho-dihydroxylated FLVs are responsible for efficient scavenging of ROS as well as inhibiting formation of free radicals. Besides FLVs, excess energy can be dissipated by non-photochemical quenching, particularly via the carotenoids of the xanthophyll cycle, which also contribute to the photoprotective function of the photosynthetic apparatus [9]. Interplay between accumulation of PheCs and carotenoids under excess light conditions is still ambiguous. In some species, anthocyanins might partially compensate for the effect of the photo-protective xanthophyll cycle. In other cases, anthocyanins appear to be an alternative, rather than compensatory, photoprotective strategy [2,10]

Temperature affects a number of physiological processes at both cell and whole plant level. The metabolic response to temperature is also at least partially regulated on transcriptional level, because the expressions of genes of initial steps of phenylpropanoid (phenylalanine ammonia lyase—PAL) and FLVs (chalcone synthase—CHS) biosynthesis pathways are often enhanced under low temperature [11,12,13,14,15], but only in the light [16]. Moreover, several related transcription factors belonging to the MYB family [11,17,18] with inductive effects on genes coding for enzymes of PheCs biosynthetic pathway are also induced by low temperature (LT). Notably, modulation of PheCs production may occur on other levels as shown on the example of multi-level regulation of CHS quantity and activity [19], which should not be neglected. There is an evidence that response of PheCs metabolism to temperature is reliant on the light signal [11]. Intriguingly, interaction of light and temperature cues leading to subsequent changes in expression of PheCs genes and related transcription factors occurs at very beginning stage of light perception, since temperature affects the photoceptors, their mutual interaction, and other molecules involved in photo-perception (e.g., phytochrome-interacting factors (PIFs), suppressor of phytochrome A-105 (SPA), and other protein partners; [20,21,22]). The typical response of plants to low temperature is enhanced production of anthocyanins [23]; however, their content in barley plants is usually very low. Thus, it seems that contrary to other plant species (e.g., *A. thaliana*), anthocyanins do not contribute significantly to photoprotection at low temperature, and they are not within the scope of this study.

Spring barley is a leading widely cultivated crop with significant food and economical importance. Domestication and breeding of barley lead to production of varieties with high yield, and the allelic diversity was reduced during these processes, which can affect resistance to abiotic stress [24]. It is therefore necessary to evaluate the mechanisms that are responsible for the resistance to environmental stressors and whether this resistance is at the expense of the growth rate. Low (LT) or high temperature (HT) may act synergistically with high irradiance (HI) to increase the susceptibility of photosystem II to photoinhibition. On the other hand, the light (at low irradiances) may serve as an effective protector against heat-induced inactivation of PSII [25]. Considering this, it is worthy to study various combinations of these two factors to assess their possible physiological consequences as they often co-act at natural conditions, e.g., during summer clear sky days (HI HT), autumn cloudy days (LI LT), winter/spring open sky (HI LT), shaded plants during summer days (LI HT). Therefore, we evaluated how a combined effect of both irradiance and temperature affect gene expression, accumulation, and metabolic profile of PheCs in relation to direct co-action of these factors and in relation to availability of their precursors originating from photosynthesis and subsequent reaction of primary metabolism. The significance of individual phenolic compounds and photosynthetic pigments is discussed in relation to their capability of ROSs scavenging.

## 2. Results

### 2.1. Photosynthetic CO_2_ Assimilation and Non-Structural Carbohydrates Content

**Figure 1:**
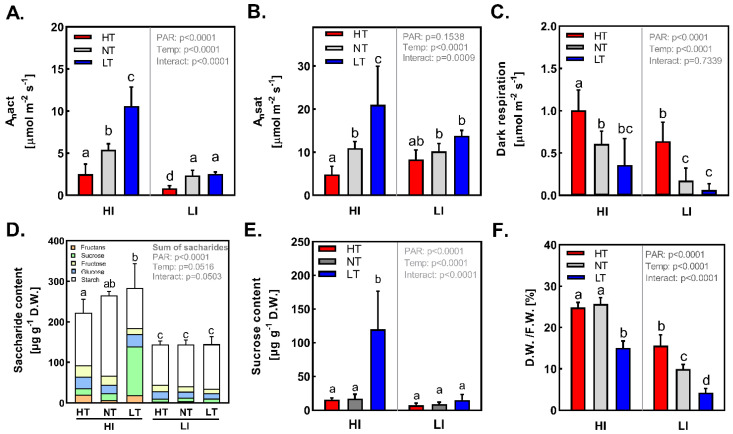
(**A**)—Actual photosynthetic CO_2_ assimilation rate (A_n_), (**B**)—CO_2_ assimilation capacity (A_nsat_), (**C**)—Dark respiration rate, (**D**)—Non-structural carbohydrates content: (fructans (Fra), sucrose (Suc), fructose (Fru), glucose (Glu), starch (Str)), (**E**)—Sucrose content, and (**F**)—Ratio of dry/fresh weight of the *Hordeum vulgare* L. cv. Bojos acclimated to the conditions varying in total irradiance and temperature. Specifications of light and temperature treatments: HI (high irradiance, 1000 µmol m^−2^ s^−1^), LI (low irradiance, 50 µmol m^−2^ s^−1^), HT (high temperature; 35 °C), NT (normal temperature, 20 °C), LT (low temperature, 12 °C); *n* = 5–6 ± SD. Figures contain results of two-way ANOVA. Treatments marked above with same letters did not significantly differ based on Tukey’s post hoc test.

The photosynthetic CO_2_ uptake by barley plants under individual treatments was estimated by measuring the actual photosynthetic CO_2_ assimilation rate (A_nact_). The total irradiance of PAR was the main factor, which significantly enhanced A_nact_ (*p* < 0.0001). In addition, the negative effect of increasing temperature was more pronounced (*p* < 0.0001) in plants acclimated to HI (Figure 1A). The results of CO_2_ assimilation capacity (A_nsat_) (Figure 1B) copied this trend in a very similar way. In the case of the dark respiration (R_d_), a marked effect of temperature was observed; HT increased while LT decreased R_d_ parameter, irrespective of irradiation (Figure 1C). The actual photosynthetic CO_2_ assimilation rates of plants acclimated to high irradiances correlated well with total leaf non-structural carbohydrate (NSC) levels that increased with decreasing temperature (Figure 1D). Interestingly, under normal temperature, starch was prevailing while under LT conditions sucrose clearly dominated in the NSC spectrum (Figure 1E). Low irradiance was limiting for NSC accumulation, irrespective of temperature.

### 2.2. Photosynthetic Pigments Content and Composition

**Figure 2:**
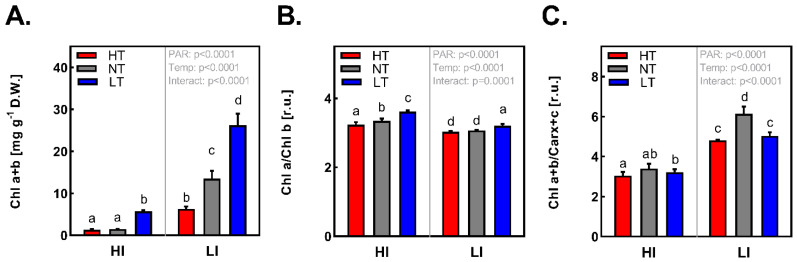
(**A**)—Content of chlorophyll a+b, (**B**)—Ratio of chlorophyll a/b, (**C**)—Ratio of total chlorophylls (a+b) and carotenoids (xanthophylls and carotenes) of *Hordeum vulgare* L. cv. Bojos in leaves acclimated to the conditions varying in total irradiance and temperature. Specifications of light and temperature treatments: HI (high irradiance, 1000 µmol m^−2^ s^−1^), LI (low irradiance, 50 µmol m^−2^ s^−1^), HT (high temperature; 35 °C), NT (normal temperature, 20 °C), LT (low temperature, 12 °C); *n* = 5–6 ± SD. Figures contain results of two-way ANOVA. Treatments marked above with same letters did not significantly differ based on Tukey’s post hoc test.

The contents of total chlorophylls (Chl a+b) per dry weight were reduced in all HI treatments (Figure 2A), suggesting acclimation of photosynthetic apparatus to excessive light [26] that is accompanied by a greater accumulation of assimilates (Figure 1D) and thus by an increased portion of dry matter under HI conditions (Figure 1F and Figure S2). Besides that, a pronounced negative effect of HT on photosynthetic pigments was observed for plants acclimated to LI. Differences in Chl a+b contents among the treatments inversely correlate with changes in D.W./F.W. (Figure 1F). Acclimation to HI is clearly demonstrated by an increase of Chl a/b ratio, indicating reduced size of light-harvesting complexes. On the other hand, HT causes rather the opposite acclimation of photosynthetic apparatus, apparently indicating an increased size of the light-harvesting system in comparison with plants acclimated to the same irradiance and LT (Figure 2B). In general, the effect of LT is more obvious. We observe a significant increase in both Chl a+b content and Chl a/b ratio. Comparison of the chlorophylls ratio and total carotenoids content (xanthophylls and carotenes) (Figure 2C) points to a tendency to increased resistance of the photosynthetic apparatus to excessive light in HI acclimated plants by increasing the amount of photoprotective carotenoids per chlorophylls. The effect of temperature is not so obvious in this case, however the highest Chl a+b/Car x+c ratio at NT indicates slightly enhanced demands on carotenoid-mediated protection in plants cultivated at both LT and HT, as compared to NT counterparts.

**Figure 3:**
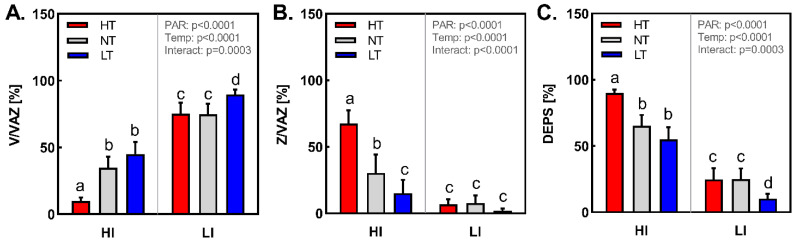
(**A**)—Ratio of violaxanthin/(violaxanthin + antheraxanthin + zeaxanthin), (**B**)—Ratio of zeaxanthin/(violaxanthin + antheraxanthin + zeaxanthin), and (**C**)—The de-epoxidation state (DEPS) of xanthophyll cycle (zeaxanthin + antheraxanthin)/(violaxanthin + antheraxanthin + zeaxanthin) in leaves of *Hordeum vulgare* L. cv. Bojos acclimated to the conditions varying in total irradiance and temperature. Specifications of light and temperature treatments: HI (high irradiance, 1000 µmol m^−2^ s^−1^), LI (low irradiance, 50 µmol m^−2^ s^−1^), HT (high temperature; 35 °C), NT (normal temperature, 20 °C), LT (low temperature, 12 °C); *n* = 5–6 ± SD. Figures contain results of two-way ANOVA. Treatments marked above with same letters did not significantly differ based on Tukey’s post hoc test.

In order to estimate the involvement of xanthophyll cycle [27] in barley leaves acclimated to different conditions, the leaves were sampled after 3 h of light period. As expected, de-epoxidation state (DEPS) of xanthophyll cycle pigments (violaxanthin (V), antheraxanthin (A), zeaxanthin (Z)) was significantly higher in HI plants in comparison to LI ones (Figure 3). Among LI plants, the lowest DEPS was observed in LI LT variant, whereas the amount of zeaxanthin (Z) was negligible at all cultivation temperatures. On the contrary, among HI plants, the highest DEPS was estimated in HI HT leaves, 90% of VAZ pool were presented as either antheraxanthin (A) or Z, whereas in HI LT and HI NT plants, DEPS was 55% and 62%, respectively. Increasing temperature significantly enhanced accumulation of Z in HI plants. Whereas in HI LT plants only about 15% of VAZ pool were fully de-epoxidized to Z, in HI HT plants Z amounted 70% of the VAZ pool. This is in agreement with previous findings that particularly the 2nd de-epoxidation step from A to Z, which needs a flip-flop of the A molecule across the membrane layer that is facilitated due to increasing fluidity of thylakoid membranes at higher temperatures [28,29]. Thus, particularly under HI HT conditions, the accumulated Z could considerably contribute to the protection of thylakoid membranes and their components by acting not only as mediator of efficient nonradiative dissipation of excitation energy [30], but also as antioxidant [31] and factor rigidifying thylakoid membranes [32], which is necessary mainly under HI HT conditions.

### 2.3. In-Vivo Determination of Epidermal Flavonoids (Epidermal UV-A Shielding)

**Figure 4:**
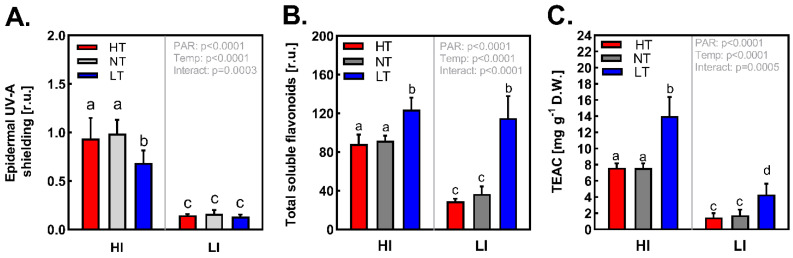
(**A**)—Epidermal UV-A shielding, (*n* = 12 ± SD), (**B**)—Content of soluble flavonoids (*n* = 5–6 ± SD), (**C**)—Antioxidant activity of soluble PheCs expressed as a TEAC (Trolox-equivalent antioxidant capacity) (*n* = 5–6 ± SD) in leaves of *Hordeum vulgare* L. cv. Bojos acclimated to the conditions varying in total irradiance and temperature. Specifications of light and temperature treatments: HI (high irradiance, 1000 µmol m^−2^ s^−1^), LI (low irradiance, 50 µmol m^−2^ s^−1^), HT (high temperature; 35 °C), NT (normal temperature, 20 °C), LT (low temperature, 12 °C). Figures contain results of two-way ANOVA. Treatments marked above with same letters did not significantly differ based on Tukey’s post hoc test.

The main factor affecting epidermal UV-A shielding during acclimation was the total irradiance. Temperature and its interaction with irradiance had a rather minor but still significant effect. Temperature affected epidermal UV-A shielding only in plants acclimated at HI conditions. Lower values were observed in case of low-temperature-treated plants (HI LT). HI NT- and HI HT-acclimated plants did not differ in epidermal UV-shielding. HI NT and HI HT plants’ coefficients for UV-A shielding efficiency were significantly higher than in HI LT plants (Figure 4A). On the contrary, under LI conditions no significant differences in UV-A shielding efficiency were observed amongst plants acclimated to HT, NT, and LT.

### 2.4. The Assessment of Total and Indivudal Soluble Flavonoids and Antioxidant Activity Assay

The total content of soluble flavonoids (FLVs) was determined as a sum of all major flavonoid peak areas in a sample per D.W. The irradiance as well as the temperature and their interaction had significant effect on the total FLV concentration (Figure 4B). The total FLV content was more than two times higher in HI compared to LI-acclimated plants; nevertheless, this effect was observed only under NT and HT conditions. On the other hand, plants acclimated to LT had the highest total content of soluble FLVs independently of acclimation irradiance.

**Figure 5:**
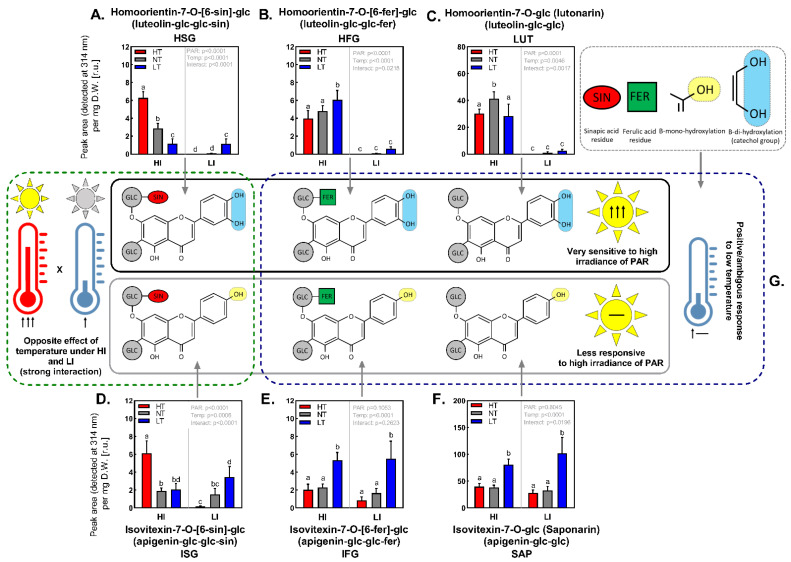
Relative content of individual flavonoids in leaves of *Hordeum vulgare* L. cv. Bojos and comparisons of their accumulation under conditions varying in total irradiance and temperature. Specifications of light and temperature treatments: HI (high irradiance, 1000 µmol m^−2^ s^−1^), LI (low irradiance, 50 µmol m^−2^ s^−1^), HT (high temperature; 35 °C), NT (normal temperature, 20 °C), LT (low temperature, 12 °C); *n* = 5–6 ± SD. (**A**)—HSG (homoorientin-7-O-[6-sin]-glc), (**B**)—HFG (homoorientin-7-O-[6-fer]-glc), (**C**)—LUT (lutonarin), (**D**)—ISG (isovitexin-7-O-[6-sinp]-glc), (**E**)—IFG (isovitexin-7-O-[6-fer]-glc), (**F**)—SAP (saponarin); generalized chemical structure reflecting the types of flavonoid aglycones and their substitutions is mentioned in brackets (glc—glucose, fer—ferulic acid, sin—sinapic acid), (**G**) Simplified chemical structures of individual flavonoids detected in spring barley secondary leaves and their different accumulation trends in response to irradiance, temperature, and various combination of these two important environmental factors. Figures contain results of two-way ANOVA. Treatments marked above with same letters did not significantly differ based on Tukey’s post hoc test.

The accumulation responses to temperature and irradiance treatments markedly differed amongst individual FLVs (Figure 5G). The major FLVs present in the extract—saponarin (SAP) and the FLV tentatively identified as isovitexin-feruoyl-glucoside (IFG)—did not respond to irradiance. However, their accumulation was significantly affected by acclimation temperature, and both HI and LI plants exhibited approximately 2× (SAP) and 3× (IFG) higher content under LT conditions, compared to respective NT and HT counterparts (Figure 5E,F).

A different pattern of both temperature and irradiance response was observed for FLVs acylated with sinapic acid, i.e., homoorientin-sinapoyl-glucoside (HSG) and isovitexin-sinapoyl-glucoside (ISG). Their accumulation was mainly stimulated by the co-action of high irradiance and high temperature. Their content was approximately 3–4× higher in HI HT compared to HI LT plants (Figure 5A,D). In plants acclimated to LI, the accumulation of HSG and ISG showed the opposite effect of temperature—stimulation by LT.

Lutonarin (LUT), the second most abundant FLV in the samples, and homoorientin-feruoyl-glucosid (HFG) responded to both factors (including their interaction). However, their quantity was mainly affected by the irradiance (Figure 5B,C). The content of these compounds was always at least 10 times higher under HI compared to corresponding LI treatments. The differences in LUT and HFG quantities due to varying acclimation temperature were not significant under LI conditions. In HI-acclimated plants, the content of HFG tended to increase with decreasing temperature, whilst LUT exhibited the most pronounced accumulation under NT.

Plants acclimated to HI exhibited markedly higher antioxidant activity of leaf extracts (3–5× times) compared to their LI counterparts. Whilst the LT increased the AOX (compared to NT) at both irradiance levels, HT did not affect the AOX (Figure 4B). In general, the AOX responded in similar way to irradiance and temperature as the total FLV content (Figure 4C). Since the main FLV of the extract (saponarin) did not respond to irradiance (Figure 5F), the increase of AOX under high irradiance treatment could be most likely attributed to intensive accumulation of HFG, LUT under HI (Figure 5B,C). To which extent examined FLVs contributed to positive effect of LT on overall AOX is not clear, yet the data suggested that HSG and ISG (Figure 5A,D) are rather less involved.

### 2.5. Analysis of Flavonoid Biosynthesis and Antioxidant Enzymes Gene Expression

**Figure 6:**
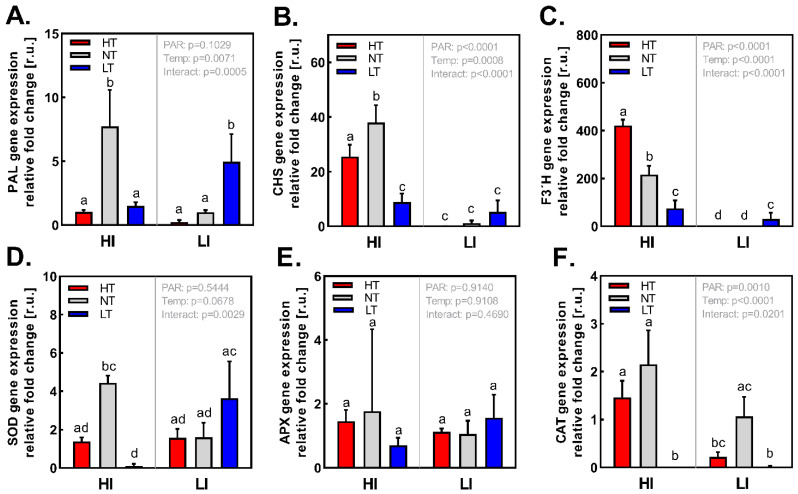
Relative gene expression of proteins involved in the production of PheCs and antioxidant enzymes in *Hordeum vulgare* L. cv. Bojos acclimated to the conditions varying in total irradiance and temperature. (**A**)—PAL (phenylalanine ammonium lyase; EC 4.3.1.24), (**B**)—CHS (chalcone synthase, EC 2.3.1.74), (**C**)—F3′H (flavonoid 3′hydroxylase; EC 1.14.14.82), (**D**)—SOD (superoxide dismutase; EC 1.15.1.1), (**E**)—APX (ascorbate peroxidase; EC 1.11.1.11), and (**F**)—CAT (catalase; EC 1.11.1.6). Specifications of light and temperature treatments: HI (high irradiance, 1000 µmol m^−2^ s^−1^), LI (low irradiance, µmol m^−2^ s^−1^), HT (high temperature; 35 °C), NT (normal temperature, 20 °C), LT (low temperature, 12 °C), *n* = 3 ± SD. Figures contain results of two-way ANOVA. Treatments marked above with the same letters did not significantly differ based on Tukey’s post hoc test.

Gene expression of phenylalanine ammonia lyase (PAL), the entry point of phenylpropanoid pathway, did not show any consistent response to either irradiance or temperature (Figure 6A). However, gene expressions of chalcone synthase (CHS) and flavanol 3-hydroxylase (F3´H), which are involved in the FLVs biosynthetic pathway, seem to be primarily regulated by light conditions (Figure 6B,C). Acclimation to HI positively affects particularly expression of *CHS* and *F3´H*, while high temperature acts synergistically with high irradiation (particularly, expression of *F3´H* was gradually upregulated with increasing temperature). Gene expression of antioxidant enzymes (Figure 6D–F), particularly superoxide dismutase (SOD) and ascorbate peroxidase (APX) were not affected by acclimation to different irradiances and temperatures, except catalase (CAT), which was positively regulated by irradiance as well as higher temperature, suggesting that a more efficient method of hydrogen peroxide detoxification is required in plants acclimated to HI HT and HI NT conditions.

## 3. Discussion

### 3.1. Photosynthetic Activity and NSC Content

From the perspective of CO_2_ assimilation rate and pigment composition of the photosynthetic apparatus, the semi-late Bojos variety of spring barley is sensitive to high temperature at the beginning of its tillering phenological phase [33]. Although measurements of PS II quantum efficiency indicated quite high thermo-tolerance of barley (Figure S3), this was not accompanied by the high intensity of photosynthetic assimilation of CO_2_ (Figure 1A). The highest content of Chl a+b in both LI LT and HI LT leaves (Figure 2A), as compared to plants acclimated at NT and HT and corresponding irradiance, suggested that barley plants rather thrive at low temperatures, however only in HI LT conditions plants also exhibited (concurrently) increase of A_nact_ parameter and higher concentration of NSC (Figure 1A,E). Conversely, at high temperature, the photosynthetic activity is reduced, particularly under high light conditions. Decrease of A_nact_ at HT is predominantly associated with reduced stomatal conductance, which limits the availability of CO_2_ for photosynthesis. In addition, the increase in dark respiration rate and photorespiration that occurs under HT condition (Figure 1C) reduces efficiency of CO_2_ assimilation rate and thus A_nact_. Photosynthetic rate under non-limiting light and CO_2_ conditions (A_nsat_) was reduced at HT compared to LT, and the effect of temperature was more pronounced at HI (Figure 1B). This suggests that, besides stomatal limitations, non-stomatal and biochemical limitations also occur at HT, most likely due to down-regulation of Rubisco activase, which is known to be heat inactivated starting at 35 °C [34,35,36].

High irradiance treatment enhanced photosynthetic activity and increased production of triose phosphates within Calvin–Benson cycle and subsequent synthesis of NSC which were more than 1.5-fold higher than under LI, irrespective of temperature (Figure 1E). Under optimum conditions (light, temperature), photosynthesis produces enough saccharides to cover all plant demands. Plants particularly precisely partition assimilated carbon among synthesis of sucrose and leaf starch to maintain constant supply of carbohydrates for sinks during light/dark day period [37]. Accordingly, in our experiments, barley plants were not accumulating carbohydrates in leaves under HI NT conditions, but rather effectively transported them towards sinks and store the surplus in the form of starch reserve. Accumulation of NSC under HI NT conditions is not associated with inhibition of either A_nact_ or A_nsat_, compared to other treatments, indicating sufficient sink capacity under HI NT conditions. Co-action of high irradiation and low temperature led to dissimilar response, leading to increase of both A_nact_ and NSC compared to NT (Figure 1A,E). In the case of barley, photosynthesis is rarely impaired by cold, but low temperatures effectively slow down phloem transport of photoassimilates and processes connected with utilization by sinks (lower sink demand) [38,39,40]. As a consequence, NSC accumulate in leaves. This phenotype with enhanced NSC, particularly sucrose accumulation, was observed under HI LT (Figure 1E,F). This pattern was observed also by Savitch et al. [41] in wheat and, according to our best knowledge, in the only study devoted to barley leaf metabolism under cold published by Sicher and Kremer [15]. Soluble carbohydrates, in turn, can cause suppression of photosynthesis; however, this acclimatory depression is disproportional and delayed for days [38,42]. Moreover, it depends on subcellular localization of soluble NSC [43]. Sucrose stored in vacuole can act as an osmolyte elevating cell water retention, and our data of lower D.W./F.W. in HI LT plants (Figure 1D) support such an assumption.

### 3.2. Effect of Irradiance and Temperature on UV-A Shielding

In accordance with previous studies [44,45], our results confirmed that PAR intensity strongly enhances the synthesis of epidermal PheCs in spring barley leaves. Such effect seems to be common for many other plant species [46,47]. This phenomenon was observed in our study regardless of acclimation temperature, i.e., the HI-acclimated plants exhibited higher values of epidermal shielding compared to their LI counterparts and the effect of temperature never exceeded the one of irradiance (Figure 4A). Many studies suggested that low growth or acclimation temperatures have the positive effect on the accumulation of leaf epidermal UV shielding (screening) PheCs [48,49] and the content of soluble leaf PheCs in general [18,50]. The extent of this effect could be significantly species-dependent [48] and modulated by other environmental conditions [49] as well as endogenous cues (current state of acclimation, developmental stage, etc.). However, in conditions used in our experiment, we observed lower values of epidermal UV-A shielding (Figure 4A) in plants acclimated at HI LT conditions (as compared to HI NT, HI HT), whilst in plants acclimated under LI conditions, UV-shielding remained at basal level revealing no significant effect of acclimation to different temperatures. The measurement of epidermal UV-A shielding presented in Figure 4A suggested that the PAR irradiance is the main factor effectively regulating epidermal shielding in barley secondary leaves, whilst temperature rather fine-tunes this leaf optical feature once synthesis of (epidermal) PheCs is activated by higher irradiances (e.g., by the initiation of photoreceptor signaling). However, as discussed below (and part 3.1.), the accumulation of soluble PheCs per D.W. was positively affected during acclimation to LT irrespective of irradiance, and by HI under normal and high temperatures (NT, HT) indicating more complex interaction of these two environmental factors on PheCs metabolism.

The contradiction (between the temperature effect on epidermal UV-shielding shown in literature and our results) may originate also in variations of leaf water content amongst treatments especially under high irradiance (Figure 1D). The total content of soluble FLVs was positively affected by the irradiance under NT and HT conditions (regardless of reference unit F.W. or D.W.). The content of soluble FLVs per F.W. correlated well with the response of epidermal UV-A shielding (compare Figure 4A and Figure S4) to different irradiances and temperatures including (unexpected) lower shielding efficiency of epidermal FLVs in HI LT-acclimated plants. On the other hand, when expressed per D.W., the content of soluble FLVs was the highest in LT treatments (under both HI and LI conditions) which is in accordance with generally accepted positive effect of low temperature on FLVs synthesis (Figure 4B). Actually, plants acclimated to LI LT accumulated almost the same or higher content of soluble FLVs per D.W. as their HI counterparts. Taking together abovementioned facts, we can conclude that the LT indeed enhanced the PheCs production (per D.W.), but significantly higher relative leaf water content in LT acclimated plants (Figure 1D) may negatively affect their concentration in vacuoles and thus reduce their UV-absorbance, which may in turn lead to lower epidermal UV-A shielding.

### 3.3. Impact of Acclimation Irradiance and Temperature on Soluble Flavonoid Profile and Antioxidant Activity

The majority of soluble PheCs detected in the barley leaf methanolic extracts belonged to the flavones FLVs sub-class (mainly apigenin and luteolin derivatives) and exhibited typical substitutions of their main (aglycone) structure such as glycosylation and acylation by hydroxycinnamic acids (sinapic or ferulic) (Figure 5G). The extracts also contained very low amounts of hydroxycinnamic acids (such as feruloyquinic acid isomers), which were not further analyzed in this study. PheCs metabolite profile of spring barley leaves observed in this study was in accordance with results published by Ferreres [51] and our previous work [45].

As expected, the higher irradiance had significant positive impact on the content of total soluble PheCs (proxied by the sum of main FLVs peak areas; for details, see Material and Methods) regardless of the used reference unit, i.e., D.W. or F.W. (compare Figure 4B and Figure S4). Intriguingly, this difference in FLVs content per D.W. between HI and LI treatment was not observed in plants acclimated at LT.

The most pronounced accumulation due to HI acclimation exhibited B-dihydroxylated FLVs LUT, HFG, and partially also HSG (not under LT) that were considerably less abundant in LI acclimated plants as compared to B-monohydroxylated FLVs. Especially LUT, being the second most abundant of detected FLVs in plants acclimated at HI, significantly contributed to positive response of the total FLVs content to HI. On the contrary, B-monohydroxylated FLVs (ISG, IFG, SAP) were rather less responsive to HI irradiance. Such change in ratio of B-mono-/B-di- hydroxylated FLVs was previously observed also in other species [1,52,53] and is often set in relation to higher demands on AOX activity of plants acclimated at HI.

As mentioned above the effect of temperature on the total FLV content was ambiguous and markedly dependent on reference unit (D.W. or F.W.). Accumulation of total FLV per DW in plants acclimated under LT (Figure 4B) could be attributed mainly to the increased contents of SAP (Figure 5F) and IFG (Figure 5E). Intriguingly, at HI conditions, FLV acylated by sinapic acid (HSG, ISG, Figure 5A,D) exhibited opposite response to temperature compared to those acylated by ferulic acid (IFG, HFG, Figure 5E,B) as well as non-acylated ones (SAP, LUT, Figure 5F,C) and were accumulated mainly under HT. At least in vitro, it was confirmed that sinapic acid revealed higher ROS scavenging potential (against DPPH^•^ radical 2,2-Diphenyl-1-picryl-hydrazil) compared to ferulic acid as well as it was more efficient in mitigation of linoleic acid autooxidation [54]. The functional consequence of this change of acylation pattern could be related to antioxidative properties of these products and their higher demand under conditions where higher ROS production is expected (HI HT). Indeed, the stronger impact of oxidative stress in plants acclimated at HI and HT, as compared to plants acclimated at HI NT and HI LT, was observed based on the fluorescence of SPY probe that is proportional particularly to the level of membrane lipid peroxidation (Figure S5). On the other hand, increase of FLVs containing sinapoyl group (HSG, ISG) caused by HI HT acclimation did not positively influence the overall AOX of leaf extracts (compare Figure 4C and Figure 5A,D). This could mean HSG and ISG serve as AOX in specific loci where their local concentration and thus AOX are significant (yet not high enough to manifest in total AOX), or that HT induced accumulation of HSG, ISG is not related to AOX at all. The reason why the temperature (under HI) changes the ratio of sinapoyl-a and feruloyl-flavonoids remains unknown.

Observed response of PheCs metabolism to PAR can be attributed to the co-action of light specific regulation mechanisms (related to photoreceptor signaling) as well as processes indirectly (non-specifically) regulated by light (e.g., through photosynthesis—ROS, assimilates, chloroplast retrograde signaling). To entangle and clarify these processes, their interaction, and to assess the importance of individual light-induced regulation mechanisms still remains a challenge for current research [45]. We suggest that the most important regulation cue leading to observed increase of total soluble FLVs is related to photoreceptor signaling and occurs mainly at the level of gene expression. In our study, the *CHS* gene (encoding for enzyme of initial step of FLV synthesis) and *F3´H* (enhancing production of B-dihydroxylated FLV) were significantly upregulated under HI (except for LT). These genes are, among other cues, under the control of UVR8 and CRY photoreceptors and their downstream signaling pathways such as COP1-HY5 [55,56,57,58]. Thus, upregulation of their expression under high irradiance of polychromatic PAR was expected. Surprisingly, we did not detect the upregulation of PAL gene under HI (except under HI NT conditions) (Figure 6A), which could indicate preferential distribution of assimilates from shikimate to phenylpropanoid pathway. Further, the inverse PAL gene expression response to HI in LT could be partially attributed to interaction of high irradiance and low temperature [12,59], which has not been clarified yet in the literature.

Despite the highest content of PheCs (per D.W.) in plants acclimated to both HI LT and LI LT conditions, we did not observe low temperature-induced expression of CHS, F3´H and PAL under HI and rather weaker positive effect of LT under LI (Figure 6A–C). This discrepancy probably originates from the fact that the expression of target genes usually responds very rapidly to environmental cues (in minutes/hours), whilst in our study we examined expression of above-mentioned genes involved in biosynthesis of PheCs in plants fully acclimated to HI LT conditions when the initial stimulation of gene expression might already fade out. On the contrary, PheCs accumulation is slower (rather in inter-day time scale), has a cumulative nature, and further PheCs can persist in plant material for a long time after removal of induction factor and probably after disappearing of initial gene expression response [60].

Concerning the response of genes related to antioxidative enzymes (SOD, APX, CAT), we did not observe any specific gene expression pattern which could be interpreted as the activation of AOX machinery by specific light-temperature treatments (at the gene expression level). The exception is the expression response of the CAT gene, which was markedly downregulated in LT (regardless the PAR level) and is positively enhanced by higher irradiance and temperature suggesting higher demands on H_2_0_2_ scavenging in such conditions (it is known that higher temperatures lead to enhanced production of H_2_O_2_, e.g., in OEC of PSII). However, the measurement of enzymatic AOX was not analyzed in this study; we presume that the trends of AOX gene activity can be markedly different from gene expression trends (especially when measured at the end of acclimation period).

To what extent the light and temperature modulate accumulation of PheCs through saccharide availability/content is still unclear. Our data suggested that the NSC content may support the PheCs production—the NSC content was markedly higher under HI at all temperature levels; moreover, we observed positive effect of LT on NSC accumulation (i.e., NSC had similar response pattern as total PheCs). We cannot present direct causative links (molecular mechanisms) between inductive factors (irradiance/temperature), NSC, and PheCs contents. However, the data indicate that positive effect of LT on photosynthetic activity and subsequently PheCs content could be due to reduced demand for primary assimilates (due to slower growth) and thus lesser sink consumption of NSC strengthened by slower phloem transport of NSC resulting in sucrose accumulation and higher availability of carbon for secondary metabolism [15]. Still, changes in NSC solely cannot easily explain fine modulation of individual PheCs unless the different sensitivity of FLV genes to individual saccharide-specific signaling cues are taken into account as proposed, e.g., by Solfanelli et al. [6]. High sucrose content could be the cause of higher PheCs content in HI LT and can be hypothetically explained by the synergy of photoreceptor and saccharide signaling. The sucrose-dependent regulation of PheCs biosynthesis related genes may occur on the gene expression level, e.g., CHS and several other genes involved in this pathway (such as dihydroxy dihydroflavonol reductase coding genes; [6]) are sensitive to sucrose signal. For example, CHS gene together with other genes is under control of MYB75 (PAP1), which responds directly to the sucrose concentration [61]. In addition, it is also documented that CHS has in the promoter region suc boxes [62], which seem to be related to suc-induced expression, and some studies suggest suc-box role as an enhancer [63] of transcription. Nevertheless, expected correlation between sucrose content and the gene expression levels of CHS or F3´H was not found in our study (compare Figure 1 and Figure 4) in the end of acclimation (explained above). Such kind of positive response was observed in case of exogenously added sucrose or saccharides accumulating mutants [6]. We assume that in the advanced state of acclimation (one-week exposure to various conditions) the production of PheCs may be positively affected by the saccharides because of their availability for PheCs synthesis rather than due to enhanced expression of related genes (gene expression, enzyme quantity, and activity may exhibit significantly different temporal patterns). Besides a direct effect of sucrose on PheCs level, an indirect regulation should be considered. It was demonstrated that exceeding the threshold level of sucrose and reduction of sink strength at low temperature cause a reduction of nitrate uptake [64,65], which is followed by accumulation of FLVs, due to downregulation of primary metabolites synthesis. Future experiments thus should be more focused on the observation of PheCs enzyme activity and its relation to saccharide content and not exclusively on regulation of gene expression, which is much more accented in the literature.

## 4. Materials and Methods

### 4.1. Plant Material and Cultivation Conditions

Spring barley plants (*Hordeum vulgare* L. cv. Bojos) were cultivated from seeds for 7 days in a growth chamber (BioLine HB1014, Heraeus Vötch—Industrietechnik, Hanau, Germany) under medium irradiance and normal temperature (MI NT; 400 µmol m^−2^ s^−1^; 20 °C) with relative air humidity of 70% in a 16/8 h light/dark regime. After that time period, plants were acclimated for another 7–8 days to six different treatments as follows: high irradiance and high temperature (HI HT; 1000 µmol m^−2^ s^−1^; 35 °C), high irradiance and normal temperature (HI NT; 1000 µmol m^−2^ s^−1^; 20 °C), high irradiance and low temperature (HI LT; 1000 µmol m^−2^ s^−1^; 12 °C), low irradiance and high temperature (LI HT; 50 µmol m^−2^ s^−1^; 35 °C), low irradiance and normal temperature (LI NT; 50 µmol m^−2^ s^−1^; 20 °C), low irradiance and low temperature (LI LT; 50 µmol m^−2^ s^−1^; 12 °C). All sampling for biochemical analysis (photosynthetic pigments, non-structural carbohydrates, AOX activity, PheCs, gene expression) were carried out on day 7, while gas exchange measurements was performed on days 7 and 8 due to greater time requirements. The temperature during the dark period was always by 20% lower as compared to the light one. Plants were cultivated in pots (6 pots per each treatment, 20 plants per pot) in the mixture of substrate for pot plants and garden substrate (Agro CS, Česká Skalice, Czech Republic) (1:1, *v*/*v*) without additional fertilizers and were regularly irrigated to avoid drought stress. All measurements were carried out on secondary leaves of 14–15 days old plants. Leaf dry weight was determined by drying the leaf segments at 105 °C for 60 min, and it was calculated as the ratio of dry weight to fresh weight (D.W./F.W.).

### 4.2. Gas Exchange Measurements

Actual photosynthetic CO_2_ assimilation rates, as well as light response curves, were measured with an open gas exchange system Li-6400XT (LI-Cor Inc., Lincoln, NE, USA) equipped with the 2 × 3 cm broadleaf chamber and integrated light source (Li-6400-02B; Li-Cor, Inc). Actual photosynthetic rate (A_nact_) was performed at the same photosynthetic photon flux density (PFFD) and temperature at which the plants of the given treatment were acclimated. Once A_nact_ was measured, the light response curves (LRC) were performed at saturated CO_2_ concentration. The first value of the LRC curve was measured at PPFD = 1500 µmol m^−2^ s^−1^, then PPFD was decreased stepwise to 0 µmol m^−2^ s^−1^. The lag between two consecutive measurements at different PPFD was 3–6 min. In order to determine the parameters CO_2_ assimilation capacity (A_nsat_) and dark respiration (R_d_), the data for photosynthesis versus irradiance were fitted according to Ögren and Evans, which provides a good description of the response of photosynthesis to light [66].

### 4.3. Non-Structural Carbohydrate and Starch Content Assesment

Approximately 100 mg of fresh leaves were harvested and immediately frozen in liquid nitrogen at the beginning of the photoperiod on the 7th day of acclimation to different light and temperature. Samples were freeze-dried (Lyovac, GT2, FINN-AQUA SANTASALO-SOHLBERG GmbH, Hürth, Germany) and dry weight was determined. For extraction, 0.5 mL 80% aqueous methanol (*v*/*v*) (p.a., Lach-Ner, Neratovice, Czech Republic) was used and samples boiled 15 min at 75 °C, then vacuum-evaporated (Concentrator plus, Eppendorf) and the residue resuspended in ultrapure water (Millipore, Merck, Darmstadt, Germany). The content of non-structural soluble carbohydrates (NSC) was determined using high-performance liquid chromatography (HPLC), isocratic pump DeltaChrom SDS 030 (Watrex, Prague, Czech Republic), flow rate 0.5 mL min^−1^, mobile phase: ultra-pure MiliQ water, 80 °C) with refractometric detection (refractometer Shodex RI-71; Spectra Physics—Newport Corporation, Irvine, CA, USA, refractive index range 1–1.75), column: IEX Ca^2+^ (SUGAR SC1011, 8.0 × 300 mm, Shodex, Suite, NY, USA). The data were evaluated using Clarity 7.2 software (DataApex, Prague, Czech Republic). The starch in pellets after the extraction of soluble carbohydrates was hydrolyzed by alpha-amylase (Type VI-B from porcine pancreas, Merck, Darmstadt, Germany, 30U/sample) and amyloglucosidase (from Aspergillus niger, Merck, Darmstadt, Germany, 60U/sample) in 0.1M Na-acetate buffer (pH 4.5), samples vacuum-evaporated and treated similar to NSC. The glucose content arisen from enzymatic splitting was measured by HPLC in the same way.

### 4.4. Photosynthetic Pigment Content and Composition

The contents of total chlorophylls (Chl a+b) and total carotenoids (Car x+c) expressed per leaf dry weight, Chl a/b and Chl a+b/Car x+c ratios were estimated spectrophotometrically using a UV-VIS absorption spectrophotometer (UV/VIS 550, Unicam, Cambridge, UK) according to the equations of Lichtenthaler [67] at wavelengths 646.8 and 663.2 nm for chlorophylls and 470 nm for carotenoids. Pigments were extracted from leaves using 100% acetone with a small addition of MgCO_3_. After centrifugation at 3468 RCF for 3 min (EBA 20, Hettich Zentrifugen, Tuttlingen, Germany), the supernatant was diluted to a final concentration of 80% aqueous acetone and filtered through a 0.22 µm PTFE filter (Whatman, UK). The composition of xanthophyll cycle pigments (V/VAZ, A/VAZ, and Z/VAZ, where V, A, and Z are violaxanthin, antheraxanthin, and zeaxanthin, respectively) were estimated by gradient reversed-phase high-performance liquid chromatography using an Agilent 1200 HPLC-DAD system (Agilent Technologies, Santa Clara, CA, USA), as described in Materová et al. [68], where mobile phases and gradient elution were used for separation as described in detail in Supplementary Figure S5.

S1A. The leaves were taken three hours after the start of the light period. The segments from the middle part of the leaf blade were sampled and stored in liquid nitrogen (77K) until pigment analysis [67].

### 4.5. UV-A Shielding

The epidermal UV-A shielding index, which reflects the relative content of FLVs located in adaxial epidermis [69], was measured using the Dualex instrument (Force A, Orsay, France). The analysis was performed at the end of the experiment (14-day-old plants, i.e., 7th day of acclimation to different light and temperature conditions) on the middle segment of secondary leaves (2–3 cm from the leaf tip). Measured plants were selected randomly from individual pots.

### 4.6. Soluble Phenolic Compounds Content Assesment

Extracts used for relative quantification and identification of soluble PheCs were prepared from middle segments of spring barley secondary leaves (100 ± 5 mg of fresh weight per sample) at the end of acclimation phase (14th day of growth). Six samples were collected per treatment 3 h after the start of the light phase. Leaves segments were homogenized using mortar and pestle in 3 ml of 40% methanol (CH_4_O, ≥99.9%, Mr = 32.04 g·mol^−1^, Sigma-Aldrich, Hamburg, Germany) with a small amount of sea sand (Penta, Czech Republic). Homogenized samples were kept for 5 min in the ultrasonic bath (Ultrasonic compact cleaner K-5LE, Kraintek, Hradec Králové, Czech Republic) at the laboratory temperature to increase extraction efficiency. Subsequently, extracts were centrifuged for 3 min at 6000 RPM (EBA20, Hettich Zentrifugen, Tuttlingen, Germany). The supernatant was made up to 3 mL with 40% methanol in a volumetric tube and subsequently filtered through 0.2 µm Teflon syringe filter (Spartan, 13/0.2 RC, Whatman, Germany) into the vials and stored in the freezer (−21 °C) before the analysis.

The semiquantitative analysis of soluble PheCs was performed on a HPLC system Agilent 1200 (Agilent Technologies, Santa Clara, USA) equipped with the UV-VIS absorption diode array detector (DAD; G1315D; Agilent Technologies, USA) and the Hypersil GOLD chromatographic column (C18, 50 × 2.1 mm, 1.9 µm; Thermo Scientific, Waltham, NJ, USA). The column compartment was heated to 30 °C. The mobile phase consisted of two acetonitrile-water solutions: mobile phase A—5% acetonitrile, B—80% acetonitrile (C_2_H_3_N, ≥99.9%, Mr = 41.05 g·mol^−1^, Sigma-Aldrich, Germany) acidified by formic acid (CH_2_O_2_, Mr = 46.03 g·mol^−1^, Sigma-Aldrich, Germany) in ratio 999:1, *v*/*v*. Gradient elution was used for separation as described in detail in Supplementary Table S1B. Flow rate of mobile phases was constant (0.3 mL min^−1^) during the whole separation process. The sample of leaf extract was injected in a volume 5 µL. For the quantification of individual PheCs in plant material, peak areas detected at 314 nm were divided by sample F.W. and subsequently by the average F.W/D.W. ratio of current treatment to obtain the relative compound content per dry weight unit (further denoted simply as D.W.).

Phenolic compounds were identified by mass spectrometer micrOTOF-QII (Bruker Daltonics, Bremen, Germany) connected to UHPLC system UltiMate 3000 (Dionex, Sunnyvale, CA, USA). The chromatographic analysis was performed the same way as in the case of the HPLC-DAD quantification procedure mentioned above (i.e., same gradient, flow, chromatographic column, temperature, sample injection, etc.). The ionization in a negative (ion) mode was conducted using electrospray ionization (ESI). Quadrupole-time of flight (Q-TOF) analyzer was used to determine the mass to charge ratio (*m*/*z*) of detected compounds in the range of 50–1500. Collision induced dissociation (CID) was used to obtain fragmentation spectra (MS2) at collision energy of 35 eV. MS and MS2 spectra were compared with those obtained by the analysis of commercially available standards (such as in case of saponarin) or with the spectra published in the literature [51]. Further, the UV-VIS absorption spectra in the range from 190 to 750 nm and elution order were considered during identification process. The in-home library comprising retention times and spectra of several luteolin and apigenin based FLVs standards similar to those present in barley leaves was used. The identity of compounds was assigned to peaks detected in UV-absorption chromatograms during quantitative HPLC-DAD analysis (based on elution order and UV-VIS absorption spectra similarity).

### 4.7. Estimation of Antioxidative Activity

Antioxidant activity of leaf extracts of soluble PheCs (the extracts were prepared according to the procedures described above in the HPLC analysis of soluble PheCs) was measured spectrophotometrically using Specord 250 UV-VIS spectrometer (Analytik Jena, Jena, Germany). Determination of antioxidant potential was based on absorbance of DPPH^●^ solution (2,2-Diphenyl-1-picryl-hydrazil, C_18_H_12_N_5_O_6_, Mr = 394.32 g·mol^−1^, Sigma-Aldrich, Schnelldorf, Germany) after reaction with extract of PheCs. Working solution of DPPH^●^ was prepared by dissolving a 7.5 mg of the substance in 100 mL of 100% methanol (CH_4_O, ≥99.9%, Mr = 32.04 g·mol^−1^, Sigma-Aldrich, Schnelldorf, Germany). Mixing 2 mL of DPPH solution with 0.5 mL of extract was followed by 10 min incubation at room temperature. Then, the sample was poured into a 6030-UV quartz cuvette (Hellma, Germany) with subsequent determination of the absorbance decrease compared to the blank at a wavelength of 515 nm and a monochromator slit optical width of 0.5 nm. The resulting value was expressed in Trolox antioxidant capacity equivalent (TEAC) by fitting the absorbance to the calibration regression equation. This regression equation was obtained by preparing the Trolox (6-hydroxy 2,5,7,8-tetramethylchroman 2-dicarboxylic acid, Sigma Aldrich, Schnelldorf, Germany) concentration series in values 0, 25, 50, 100, 200, and 300 µM. Absorbance of the samples expressed in TEAC value was related to F.W. of the secondary leaves and adjusted to D.W. index.

### 4.8. Relative Gene Expression Analysis Using RT-qPCR

Approximately 50 mg of plant tissue per sample was taken for analysis and immediately frozen in liquid nitrogen. Homogenization using mortar and pestle followed. Resulting soft powder was washed into nuclease-free Eppendorf tube by adding 500 µL of TRIzol (Sigma Aldrich, St. Louis, MO, USA Cat No. T9424), and further processed as described in [45] using Turbo DNAse Free TM kit (Ambion, Austin, TX, USA, Cat No. AM 1907) to get rid of DNA traces and First Strand cDNA kit (Thermo Scientific, Waltham, NJ, USA, Cat No. K1612) for reverse transcription. qPCR reaction followed as described in [45] using EliZyme Green mix Add ROX (Elisabeth Pharmacon, Brno, Czech Republic, Cat No. EZ4614) and primer sequences (Thermo Fisher Scientific, Waltham, NJ, USA) from the available literature [70,71,72,73,74,75]. cDNA was subjected to amplification by the LC480 Instrument (Roche Diagnostics GmbH, Mannheim, Germany). Gained data were processed as described in Livak and Schmittgen (2001) [76]. Alpha tubulin was chosen as reference gene and differential expression was calculated relative to LI NT treatment.

### 4.9. Data Visualisation and Statistical Analysis

Statistical processing and data visualization were performed in GraphPad Prism software (version 9.1.2.226, GraphPad Software, San Diego, CA, USA); additional schemes shown in Figure 5 were created in Microsoft PowerPoint (Microsoft 365, Microsoft, Redmond, WA, USA). The data normality was verified by Shapiro–Wilk test and QQ plot. To assess the effects induced by acclimation treatments differing in PAR irradiance and temperature (and their combinations) two-way ANOVA was used (resulting *p*-values belonging to individual acclimation factors and to their interaction are shown in the upper-right corner of each figure). Subsequently, Tukey’s post hoc test was applied for the multiple-group comparisons; values which did not differ significantly were labeled by the same letter (above corresponding bars). For most of the analysis (NSC, photosynthetic pigments, soluble PheCs and antioxidative activity) 5–6 samples of fresh leaves were taken for each treatment except UV-A shielding, which was based on 12 measurements. Gas exchange measurements were carried out on 6 samples of intact leaves for each treatment. PCR reaction was performed in three biological and three technical replicates (in total 9 reactions) per treatment.

## 5. Conclusions

In summary, photosynthetic activity as well as the production of non-structural carbohydrates of barley seedlings were positively affected by increasing PAR, which was accompanied by adjustment of the photosynthetic apparatus proved by changes in photosynthetic pigments composition. On the contrary, an increase in temperature caused down-regulation of photosynthesis and greater accumulation of zeaxanthin, which acts as significant excitation energy dissipator and antioxidant. We observed a strong positive effect of high PAR and low temperature on the total soluble FLV content. Total FLV production was particularly enhanced by low temperature under low-PAR conditions. Such response of FLV content was partially supported by higher availability of assimilates for secondary metabolism which is implied by higher CO_2_ assimilation rates and accumulation of non-structural carbohydrates. However, the response of individual FLVs to light and temperature treatments distinctly varied, e.g., based on their B-hydroxylation and acylation patterns. This suggests specific physiological functions of these compounds and precise tuning of FLV biosynthetic pathway by co-action of examined environmental stimuli (mainly on the gene expression level). Concerning the physiological significance, the changes in flavonoid profile induced by high PAR and low temperature led to higher AOX activity of leaf extracts and enhanced UV-shielding (only in case of high PAR), which may in turn make barley plants more tolerant to photo-oxidative stress.

## Data Availability

Processed and derived data are available from the corresponding authors (J.N and V.Š.) on request.

## Supplementary Materials

The following supporting information can be downloaded at: https://www.mdpi.com/article/10.3390/ijms24032427/s1.
