# Supplementary material for: Light Drives and Temperature Modulates: Variation of Phenolic Compounds Profile in Relation to Photosynthesis in Spring Barley
Source: Int J Mol Sci. 2023 Jan 26;24(3):2427. doi: 10.3390/ijms24032427 (PMC9916737; doi:10.3390/ijms24032427)
Supplement: Supplementary file 1 [file ijms-24-02427-s001.zip › ijms-2069927-SI.pdf]

*Supplementary material*

# Light drives and temperature modulates: Variation of phenolic compounds profile in coordination with photosynthesis in spring barley

**Daniel Vrábl [1,†], Jakub Nezval [1,†,*], Radomír Pech [1], Adriana Volná [1], Petra Mašková [2], Jan Pleva [1], Nikola Kuzniciusová [1], Michaela Provazová [1], Michal Štroch [1,3] and Vladimír Špunda [1,3,*]**

1 Department of Physics, Faculty of Science, University of Ostrava, 710 00 Ostrava, Czech Republic; daniel.vrabl@osu.cz (D. V.); jakub.nezval@osu.cz (J. N.); radomir.pech@osu.cz (R. P.); adriana.volna@osu.cz (A.V.); h.pleva@seznam.cz (J.P.); nikikuzmova@gmail.com (N.K.); mprovazova@seznam.cz (M.P.)
2 Department of Experimental Plant Biology, Faculty of Science, Charles University, 128 00 Prague 2, Czech Republic; petra.maskova@natur.cuni.cz (PM.)
3 Global Change Research Institute, Czech Academy of Sciences, 603 00 Brno, Czech Republic
* Correspondence: vladimir.spunda@osu.cz (V.Š.), jakub.nezval@osu.cz (J.N)
† These authors contributed equally to this work.

**Content:**

1. Figure S1: Simplified scheme of flavonoid production in plants and its interconnections with primary metabolism.
2. Table S1: Gradient of mobile phases used for HPLC based PheC (A) and pigment (B) separation.
3. Figure S2: Leaf dry mass per unit of leaf area (LMA) of secondary leaves of *Hordeum vulgare* L. Bojos acclimated to the conditions varying in temperature.
4. Figure S3: The maximum quantum efficiency of PSII determined based on the measurement of $F_v/F_m$ chlorophyll fluorescence parameter.
5. Figure S4: Total PheC content relative to leaf fresh weight (F.W.) of *Hordeum vulgare* L. cv. Bojos acclimated to the conditions varying in total irradiance and temperature.
6. Figure S5: Content of lipid peroxides in secondary leaves of *Hordeum vulgare* L. cv. Bojos acclimated to the conditions varying in temperature.

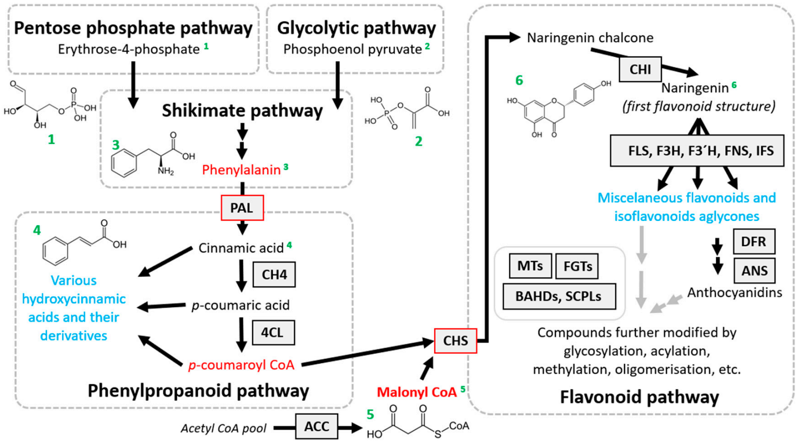

**Figure S1.** ~~Fig. S1.~~: Simplified scheme of flavonoid production in plants and its interconnections with primary metabolism. The production of flavonoids requires substrates/precursors originating from primary metabolism—i.e. Pentose phosphate pathway (Phosphoenol pyruvate; 1), Glycolytic pathway (Erythrose-4-phosphate; 2), and Acetate pathway (Malonyl CoA; 5; produced from Acetyl CoA). Phosphoenol pyruvate and Erythrose-4-phosphate are utilised within the Shikimate pathway resulting in production of Phenylalanine (3). Phenylalanine is then metabolised by the initial enzyme of Phenylpropanoid pathway—Phenylalanine ammonium lyase (PAL) to Cinnamic acid (4). Subsequently, Cinnamic acid is metabolised in Phenylpropanoid pathway by CH4 and 4CL to *p*-coumaric CoA, which is an important precursor for flavonoid production. Yet, the Phenylpropanoid pathway is responsible for the production of plethora of phenolic acids (mainly hydroxycinnamic acids and their derivatives), which can serve as precursors for the synthesis of other important phenolic compounds such as monolignols, lignin or hydrolysable tannins, etc. The first reaction of Flavonoid pathway is mediated by Chalcone synthase (CHS), which uses *p*-coumaric CoA and Malonyl CoA (5) as substrates to produce Naringenin chalcone. This compound is later isomerised by Chalcone isomerase (CHI) to Naringenin (6)—first flavonoid aglycone created within this pathway. From the Naringenin flavonoid pathway starts to branch out involving many enzymes such FLS, F3H, F3´H, FNS, IFS, which specifically modify flavonoid main structure and their hydroxylation pattern leading to production of many various flavonoid aglycones belonging to different flavonoids sub-classes (e.g., flavones, flavonols, isoflavonoids). Importantly, the latest steps of flavonoid pathway (involving ANS, DFR enzymes) lead to production Anthocyanidins. However, the structural variability of flavonoids is further increased by plethora of possible substitutions of the aglycone structure (glycosylation, acylation, methylation) and also by possible oligomerisation, such modification are catalysed by, e.g., MTs, FGTs, BAHDs, SCPLs. CH4—cinnamic acid 4-hydroxylase; 4CL—4-coumarate:CoA ligase; ACC—Acetyl-CoA carboxylase; FLS—flavonol synthase; F3H—flavanone 3 hydroxylase; F3´H—flavonoid 3´-hydroxylase; FNS—flavon synthase; IFS—isoflavone synthase; ANS—anthocyanidin synthase; DFR—dihydroflavonol 4-reductase; CoA—coenzyme A; MTs—methyl-transferases; FGTs—flavonoid-glycosyl transferases; BAHDs, SCPLs—acyl-transferases.

**Formatted:** MDPI_5.1_figure_caption

**Table S1:** Gradient of mobile phases used for HPLC based photosynthetic pigments (A) and PheCs (B) separation.

| A | | | |
|---|---|---|---|
| **Time [min]** | **ACN:MeOH:TRIS (241:30:1 v/v) [%]** | **MeOH:Hexane (4:1 v/v) [%]** | **MeOH [%]** |
| 11 | 100 | 0 | 0 |
| 13 | 0 | 100 | 0 |
| 20 | 0 | 100 | 0 |
| 21 | 0 | 0 | 100 |
| 25 | 0 | 0 | 100 |

| B | | |
|---|---|---|
| **Time [min]** | **5% ACN [%]** | **80% ACN [%]** |
| 0 | 100 | 0 |
| 2 | 95 | 5 |
| 10 | 80 | 20 |
| 15 | 60 | 40 |
| 18 | 20 | 80 |
| 22 | 0 | 0 |

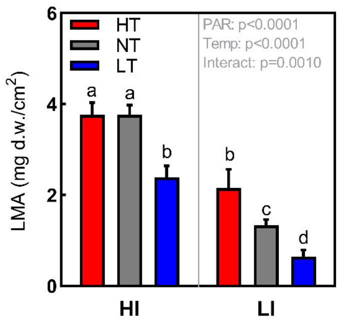


**Figure S2**: Leaf dry mass per unit of leaf area (LMA) of secondary leaves of *Hordeum vulgare* L. Bojos acclimated to the conditions varying in temperature. Specifications of light and temperature treatments: irradiance 400 µmol m$^{-2}$ s$^{-1}$, HT (high temperature; 35°C), NT (normal temperature, 20°C), LT (low temperature, 12°C); $n$ = 5-6 ± SD. One-way ANOVA confirmed significant effect of temperature (p<0.0001). Treatments marked above with the same letters did not significantly differ based on Tukey's *post-hoc* test.

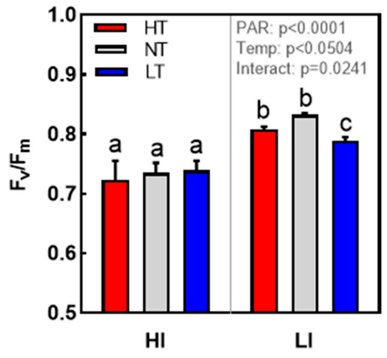


**Figure S3**: The maximum quantum efficiency of PSII determined based on the measurement of $F_v/F_m$ chlorophyll fluorescence parameter. Measurement was performed on the middle part of secondary leaves which were dark-adapted using leaf-clips for 2h in conditions specific for each individual treatment (i.e., the whole plant with a clip on secondary leaf remained in current light and temperature conditions in the growth chamber before the analysis). HI (high irradiance, 1000 µmol m$^{-2}$ s$^{-1}$), LI (low irradiance, 50 µmol m$^{-2}$ s$^{-1}$), HT (high temperature; 35°C), NT (normal temperature, 20°C), LT (low temperature, 12°C); $n$ = 5-6 ± SD. Figure contains results of two-way ANOVA. Treatments marked above with same letters did not significantly differ based on Tukey's *post-hoc* test.

**Formatted:** Font: Italic

**Formatted:** Font: Italic

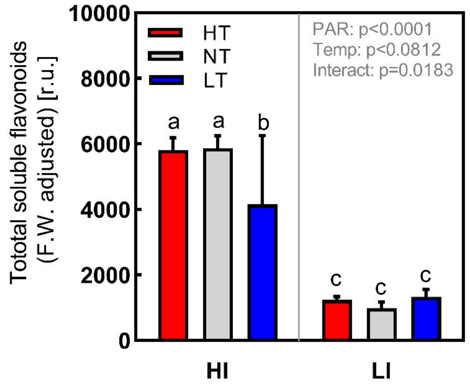


**Figure S4**: Total PheC content relative to leaf fresh weight (F.W.) of *Hordeum vulgare* L. cv. Bojos acclimated to the conditions varying in total irradiance and temperature. Specifications of light and temperature treatments: HI (high irradiance, 1000 µmol m$^{-2}$ s$^{-1}$), LI (low irradiance, 50 µmol m$^{-2}$ s$^{-1}$), HT (high temperature; 35°C), NT (normal temperature, 20°C), LT (low temperature, 12°C); *n* = 5-6 ± SD. Figure contains results of two-way ANOVA. Treatments marked above with same letters did not significantly differ based on Tukey's *post hoc* test.

**Formatted:** Font: Italic

HI—high irradiance of PAR (1000), HT—high temperature (35 °C), NT—normal/medium temperature (20°C), LT—low temperature (12°C)

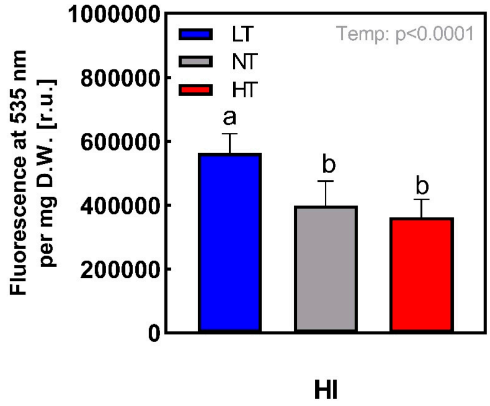


**Figure S5**: Content of lipid peroxides in secondary leaves of *Hordeum vulgare* L. cv. Bojos acclimated to the conditions varying in temperature. Specifications of light and temperature treatments: irradiance 400 µmol m$^{-2}$ s$^{-1}$, HT (high temperature; 35°C), NT (normal temperature, 20°C), LT (low temperature, 12°C); *n* = 5-6 ± SD. Peroxylated lipids were determined using a 2 µM solution of Spy-LHP fluorescence probe dissolved in ethanol. Spy-LHP solution in a volume 1.8 ml was mixed with 0.2 ml of leaf extract prepared in MES-NaOH buffer. Samples were centrifuged at 12000 g for 2

min. The fluorescence intensity of the supernatant was measured using a Fluorolog-3 at excitation/emission wavelength 488/535 nm and optical slit width set up to 1.8 nm for excitation and emission. Fluorescence signal was adjusted to mg FW. One-way ANOVA confirmed significant effect of temperature ($p$<0.0001). Treatments marked above with same letters did not significantly differ based on Tukey's post hoc test.

**Commented [TH1]:** Font is different here, please verify.

**Commented [DV2R1]:** It has been changed.
